# Leishmaniasis in Northern Morocco: Predominance of* Leishmania infantum* Compared to* Leishmania tropica*

**DOI:** 10.1155/2019/5327287

**Published:** 2019-08-08

**Authors:** Maryam Hakkour, Mohamed Mahmoud El Alem, Asmae Hmamouch, Abdelkebir Rhalem, Bouchra Delouane, Khalid Habbari, Hajiba Fellah, Abderrahim Sadak, Faiza Sebti

**Affiliations:** ^1^Laboratory of Zoology and General Biology, Faculty of Sciences, Mohammed V University in Rabat, Rabat, Morocco; ^2^National Reference Laboratory of Leishmaniasis, National Institute of Hygiene, Rabat, Morocco; ^3^Agronomy and Veterinary Institute Hassan II, Rabat, Morocco; ^4^Laboratory of Microbial Biotechnology, Sciences and Techniques Faculty, Sidi Mohammed Ben Abdellah University, Fez, Morocco; ^5^Faculty of Sciences and Technics, University Sultan Moulay Slimane, Beni Mellal, Morocco

## Abstract

In Morocco,* Leishmania infantum* species is the main causative agents of visceral leishmaniasis (VL). However, cutaneous leishmaniasis (CL) due to* L. infantum* has been reported sporadically. Moreover, the recent geographical expansion of* L. infantum* in the Mediterranean subregion leads us to suggest whether the nonsporadic cases of CL due to this species are present. In this context, this review is written to establish a retrospective study of cutaneous and visceral leishmaniasis in northern Morocco between 1997 and 2018 and also to conduct a molecular study to identify the circulating species responsible for the recent cases of leishmaniases in this region. Data concerning leishmaniases cases were collected from the Epidemiology and Disease Control Directorate from 1997 to 2018. Human samples obtained from peripheral laboratories were examined using PCR-ITS1 method. The ITS1 products were subjected to digestion with the restriction endonuclease Mn1-I. Between 1997 and 2018, a total of 1,255 cases of cutaneous and visceral leishmaniasis were recorded in Tangier-Tetouan-Al Hoceima Region, i.e., 1.56% of the reported cases in Morocco (1,255/80,299). Concerning the geographical study covering the period 2007-2018, 79.5% (105/132) of the sectors were affected by leishmaniases. The molecular results showed that Humans were found to be infected with the* L. infantum* species with a high infection rate compared to* L. tropica* infection. Moreover, molecular characterization using ITS1 PCR-RFLP showed that the density of* L. infantum* was significantly higher (n = 68/81; 84%) than that of* L. tropica* (n = 13/81; 16%) (*P*-value 9.894e-10). While regarding visceral leishmaniasis,* L. infantum* was the only species responsible of this form. These findings of this study showed the emergence of* L. infantum* in Morocco and suggest that this species might be more prevalent than previously thought. Furthermore, the molecular determination of* L. infantum* will be helpful for control strategies by taking into consideration the reservoir of this species.

## 1. Introduction

Leishmaniasis is a parasitic disease that affects both humans and animals and is caused by flagellated protozoa belonging to the genus* Leishmania *[1]. These unicellular protozoa are usually transmitted by the bite of female phlebotomine sand flies (*Diptera*,* Psychodidae*) [2]. It is known that more than 20 species of* Leishmania* infect mammals. They are responsible for two forms of leishmaniasis depending on the location of parasites in mammalian tissues, namely, visceral and cutaneous forms. The outcome of the infection depends on the species responsible and the immune responses of the host [3].

Human cutaneous leishmaniasis (CL) is caused by most* Leishmania* species of the subgenus* Leishmania*. In Maghreb area, including Morocco, three major species responsible for this disease are* Leishmania major*,* L. tropica*, and* L. infantum *[4], the latter being more often associated with visceral leishmaniasis. The transmission is zoonotic for* L. major *with a wild animal reservoir [5], and rather anthroponotic for* L. tropica*. In fact, the isolation of this species (MON-102 and MON-113) from dog suggests that this mammal (dog) could be implicated as the secondary reservoir host in the life cycle of* L. tropica*. Nevertheless, the few sporadic cases viscerotropic do not confirm that the dog is the principal host and the life cycle is zoonotic [6–8]. In some parts of the Maghreb,* L. Killicki* has also been proven to be responsible for zoonotic CL, particularly in Tunisia [9]. Concerning* L. infantum*, studies carried out in Maghreb countries isolated this species from dog suggesting it as the reservoir of this disease [10].

Regarding visceral leishmaniasis (VL),* Leishmania infantum* is the single agent responsible for this human form in the Mediterranean basin [11, 12]. However, Ready* et al*. suggested that any parasite responsible for cutaneous leishmaniasis can visceralize [13] (e.g.,* Leishmania tropica*, which causes CL cases, has been proven as the agent responsible for human VL and canine leishmaniasis (CanL) in Turkey [14].

In Morocco, the epidemiological situation of leishmaniasis as well as the distribution of* Leishmania *species varies. Two different eco-epidemiological entities have been known in the past: CL due to* L. major* also called wet or rural form and CL due to* L. tropica* also known as dry or urban form. However, for* L. infantum,* it was responsible for sporadic cases [4]. The recently published work revealed a new distribution of species by province. Indeed, many studies reported that CL cases in south-east of Morocco were caused essentially by* L. major *but* L. tropica *is also present [15–17]. However, in the Southwestern,* L. tropica* is the only circulating species [18–20]. In the center of the kingdom,* L. tropica* is being the only agent responsible for CL cases in some provinces [21, 22]. In others, this species was isolated beside few cases of* L. infantum *[23, 24]. Approaching north, limited cases of* L. infantum* have been also isolated with the presence of major cases of* L. tropica *[25].

Nevertheless, epidemiological data on leishmaniases are missing in the northern region. So, in order to give a general overview of the new distribution of CL species in Morocco, it is essential to carry out molecular investigations in the Northern provinces which have never been started and which remain until nowadays unknown.

The purpose of this study is to establish epidemiological data on leishmaniasis in seven provinces located in the extreme north of Morocco during the 21-year period 1997-2018 and to characterize the parasite species responsible for recent cases of leishmaniasis.

## 2. Material and Methods

### 2.1. Study Area

**Figure 1:**
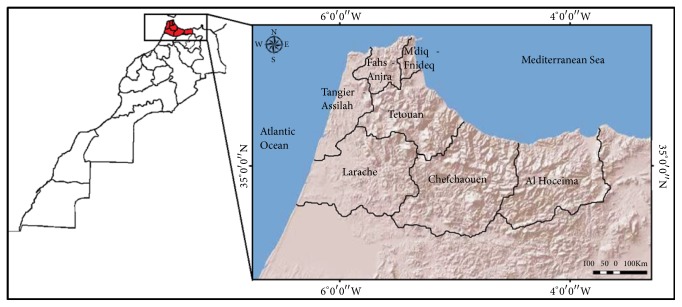
The study area.

This study was established in Tangier-Tetouan-Al Hoceima Region belonging to the northwestern section (35° 46′ 00′′ N, 5° 48′ 00′′ W) and concerns Al Hoceima, Chefchaouen, Larache, Tangier-Assilah, Tetouan, M'diq-Fnidq, and Fahs-Anjra Provinces. This region is bordered to the north by the Strait of Gibraltar and the Mediterranean Sea, to the west by the Atlantic Ocean, to the south-west by the Rabat-Sale-Kenitra Region, to the south by the Fes-Meknes Region, and to the east by the Oriental Region (Figure 1). Climatically, this region is characterized by a Mediterranean climate [26]. Moreover, in terms of tourism, its exceptional location with its two maritime facades and its roots in history and cultural diversity predispose it to occupy a favorite place as a tourist destination.

The Tangier-Tetouan-Al Hoceima Region extends over a surface of 17,262 km^2^ (2.43% of the national territory) and has a total of 3,556,729 inhabitants with a density of 206 inhabitants per km^2^ [27].

### 2.2. Diagnosis

#### 2.2.1. Microscopic Confirmation

The molecular characterization has concerned all smears received in the National Reference Laboratory for Leishmaniasis (NRLL) at the National Institute of Hygiene (NIH-Rabat, Morocco) between 2016 and 2017 (89/119). The remaining cases were not received and others were defective. These smears were sent by the peripheral laboratories in order to confirm and control their positivity. Only confirmed positive cases were included in this study.

Concerning 2018, the number of smears received was not representative compared to the number declared. As a result, these cases were excluded from the molecular study.

#### 2.2.2. DNA Extraction

The DNA extraction was performed with the Qiagen Blood and Tissue kit (Hilden, Germany) respecting the usage protocol provided by the manufacturer with minor modifications (Proteinase K was incubated for 1H at a temperature of 56°C) [28].

#### 2.2.3. ITS1 PCR-RFLP Analysis

The internal transcribed spacer (ITS1) region was amplified using the pairs of primers LITSR (5′-TGATACCACTTATCGCACTT-3′) and L5.8S (5′-CTGGATCATTTTCCGATG-3′). ITS1-PCR products were digested with the restriction endonuclease Mn1-I [8, 25, 29, 30]. Reference strains of* Leishmania tropica* (MHOM/MA/2010/LCTIOK-4),* Leishmania infantum* (MHOM/MA/1998/LVTA), and* Leishmania major* (MHOM/MA/2009/LCER19-09) were used as positive controls.

### 2.3. Data Collection

In 1997-2018, a total of 1,255 leishmaniases cases were the subject of an epidemiological study. Data on human cases were obtained from the Epidemiology and Disease Control Directorate [31]. The data are the result of passive surveillance based on the notifications recorded by the medical staff of the provincial laboratories of Tangier-Tetouan-Al Hoceima Region who reported each case to the Epidemiology Department of the Ministry of Health. The database contains all patient data, including sex, age, and place of residence.

The study area is known to be moderately affected by CL and highly affected by VL as well. In fact, in Morocco, the provinces considered to be strongly affected by CL recorded a total of between 10,500 and 1,000 cases during this period (such as Errachidia Province, Zagora Province, and Chichaoua Province). Provinces classified as moderately affected recorded a total between 1,000 and 100 (such as Beni Mellal Province, Settat Province, and Driouch Province). Provinces with fewer than 100 cases are considered to be slightly affected (such as Guercif Province, Agadir Province, and Tiznit Province) [32]. About visceral form, the provinces noted more than 100 cases are considered highly infected. Provinces with 100-10 cases are known to be moderately affected. Provinces registering less than 10 cases are poorly affected.

Concerning the geographical study, all data about autochthonous patients presenting the clinical symptoms of leishmaniasis during 2007-2018 were collected from health centers and infrastructure services of ambulatory actions provincial of the study provinces.

### 2.4. Statistical Analysis

Statistical analysis was performed using software R version 3.3.3.

## 3. Results

### 3.1. Molecular Diagnosis

A total of 89 slides were analyzed by ITS1 PCR-RFLP. This total is distributed over the seven Provinces: 38 CL slides in Larache Province, 21 CL/5VL slides in Al Hoceima Province, 10 CL / 1VL slides in Tetouan Province, 5 CL slides in Chefchaouen, 3 CL slides in Fahs-Anjra, 2 CL / 2VL slides in Tangier-Assilah, and 2 CL slides in Mdiq-Fnidq (Table 1).

**Figure 2:**
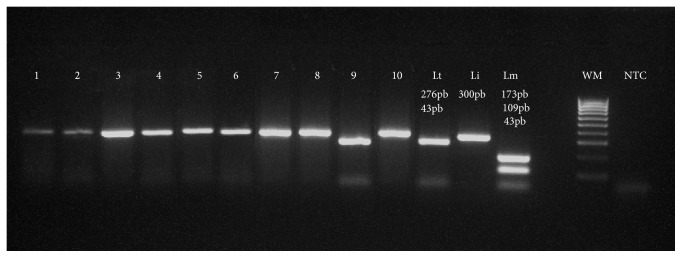
Application of analysis method ITS1 PCR-RFLP on positive slides of Leishmania in northern region. Lanes 1-8 and 10:* L. infantum*; Lanes 9:* L. tropica*; lane WM, weight marker 100 bp. Positive controls: Lt,* L. tropica*; Li,* L. infantum*; Lm,* L. major*; NTC, negative control.

The results of this molecular characterization show the coexistence of* L. infantum* and* L. tropica* responsible of cutaneous form with a predominance of* L. infantum* species (*P*-value 9.894e-10) while* L. infantum* is the only species responsible of visceral form (Figure 2) (Table 2).

**Figure 3:**
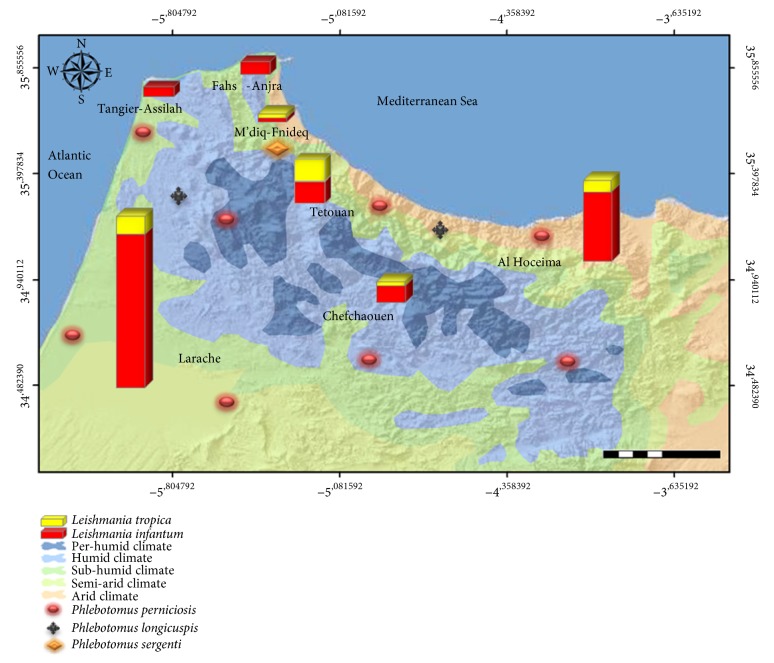
Molecular results of the circulating species in northern region according to the bioclimatic zone.

The distribution of* Leishmania* species is directly associated with the presence of sandflies species.  Figure 3 summarizes the results of molecular identification in association with the repartition of sandflies species [33] according to their bioclimatic stage preferences (http://www.water.gov.ma/ressources-en-eau/presentation-generale/).

### 3.2. Epidemiology of Leishmaniasis in Tangier-Tetouan-Al Hoceima Region

#### 3.2.1. Temporal Distribution of VL and CL Cases in Tangier-Tetouan-Al Hoceima Region

According to the Moroccan Ministry of Health, between 1997 and 2018, among the total declared (1,255) of VL and CL cases, 44.2% (n=555/1,255) have been noted as VL cases in this region.

**Figure 4:**
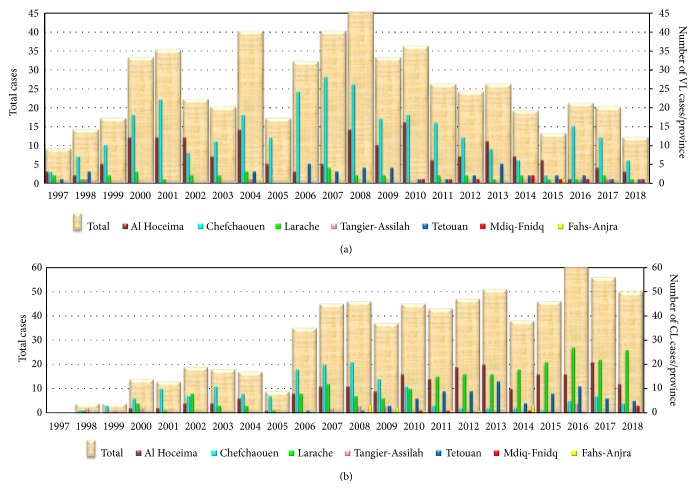
Temporal distribution of VL and CL cases. (a) Visceral leishmaniasis cases recorded between 1997 and 2018 in Tangier-Tetouan-Al Hoceima region. (b) Cutaneous leishmaniasis cases recorded between 1997 and 2018 in Tangier-Tetouan-Al Hoceima Region.

Approximately half of the total VL cases (300/555 = 54.05%) were registered in Chefchaouen Province with an average incidence of 2.86. In Al Hoceima Province, an average incidence of 2.07 per year of cases were noted (29.73% (n=165/555)) (Figure 4). As for them, Tetouan Province, Larache Province, Mdiq-Fnidq Province, Tangier-Assila Province, and Fahs Anjra Province have registered, respectively 7.20% (n=40/555), 6.49% (n=36/555), 1.44% (n=8/555), 0.72% (n=4/555), and 0.36% (n=2/555) of cases.

About CL cases, a total of 700 cases were recorded and distributed as follows: 32.14% (n=225/700) in Larache Province, 28.86% (n=202/700) in Al Hoceima Province, 23.28% (n=163/700) in Chefchaouen Province, 10.86% (n=76/700) in Tetouan Province, 2.43% (n=17/700) in Tangier-Assila, 1.57% (n=11/700) in Fahs-Anjra Province, and 0.85% (n=6/700) in Mdiq-Fnidq Province (Figure 4).

#### 3.2.2. Geographical Study of Leishmaniasis Cases in Tangier-Tetouan-Al Hoceima Region

The geographical study showed that 79.5% (105/132) of the sectors were affected by leishmaniasis in this study area between 2007 and 2018.

**Figure 5:**
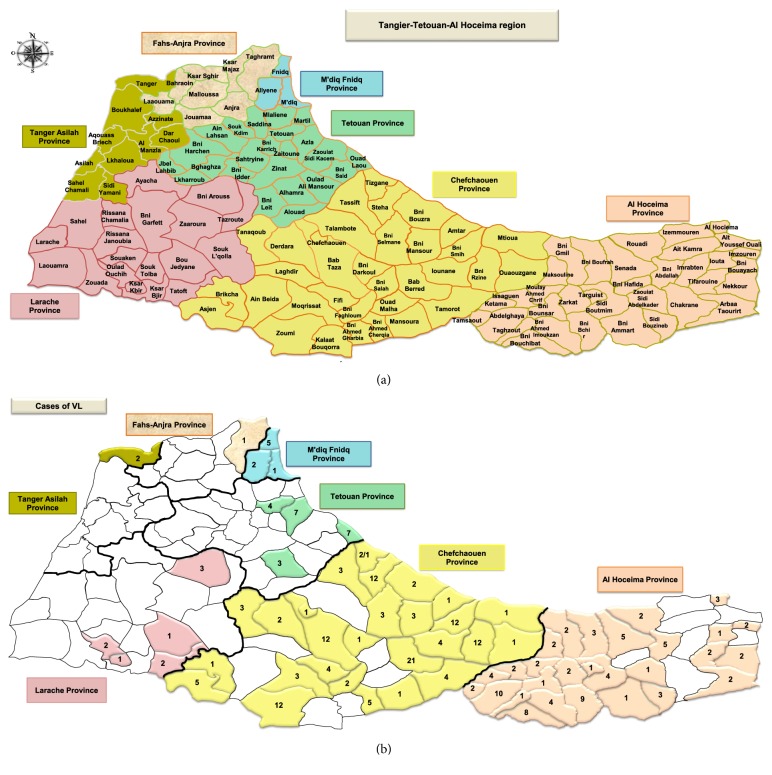
Geographical distribution of visceral leishmaniasis in Tangier-Tetouan-Al Hoceima Region (2007-2018). (a) Tangier-Tetouan-Al Hoceima region and its districts. (b) Geographical distribution of VL cases in Tangier-Tetouan-Al Hoceima Region (2007-2018).

**Figure 6:**
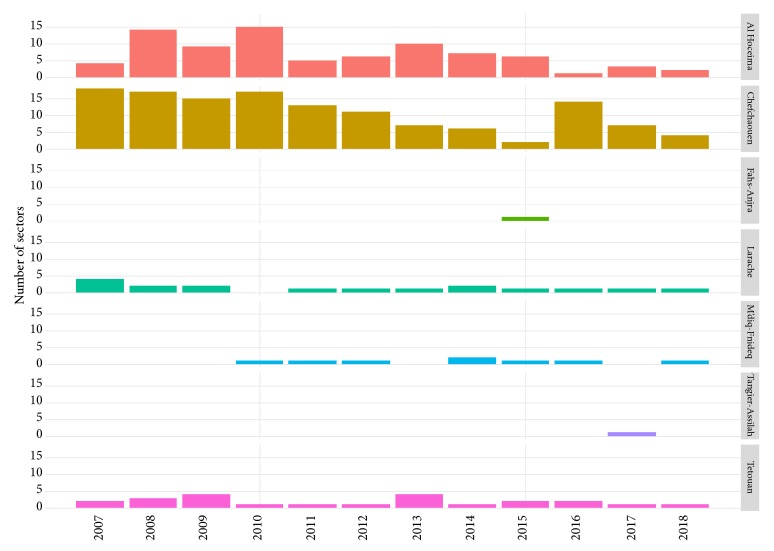
Number of affected sectors by VL depending to years in Tangier-Tetouan-Al Hoceima Region (2007-2018).

Regarding visceral leishmaniasis, a percentage of 52.3 (69/132) sectors were affected during this period (Figure 5).  Figure 6 shows a plot number of affected sectors by VL in each province per year with the majority of cases 80% (n = 28/35) noted in Al Hoceima Province. In brief, the spatial distribution of cases during the study period in this region has shown a remarkable spatial extension of VL within these provinces. In fact, there is not a concentration and a prioritization of the sectors on the other. Furthermore, it is important to note that these sectors are not regularly affected. Indeed, some sectors have been touched once or twice during this period and others have been affected yearly but distributed in different localities of the sector.

**Figure 7:**
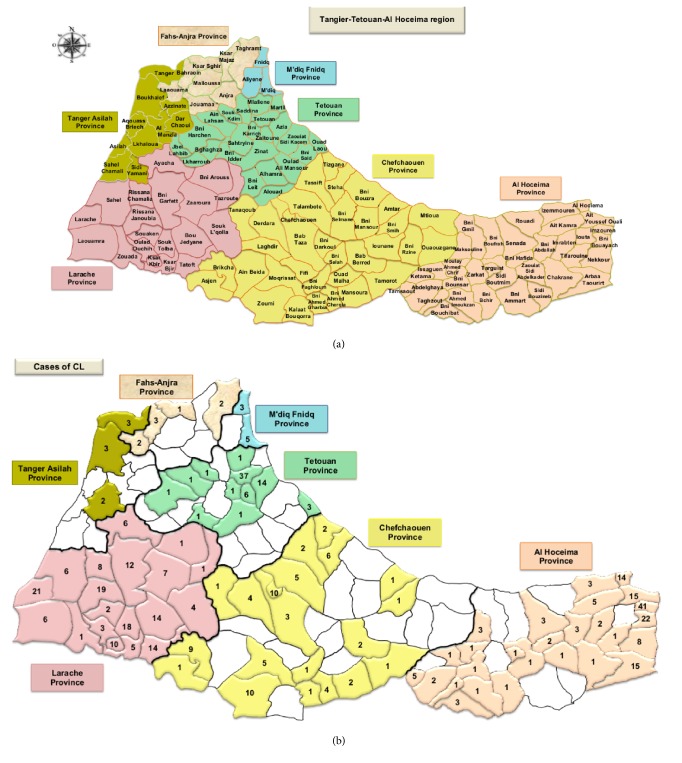
Geographical distribution of cutaneous leishmaniasis in Tangier-Tetouan-Al Hoceima Region (2007-2018). (a) Tangier-Tetouan-Al Hoceima region and its districts. (b) Geographical distribution of CL cases in Tangier-Tetouan-Al Hoceima Region (2007-2018).

**Figure 8:**
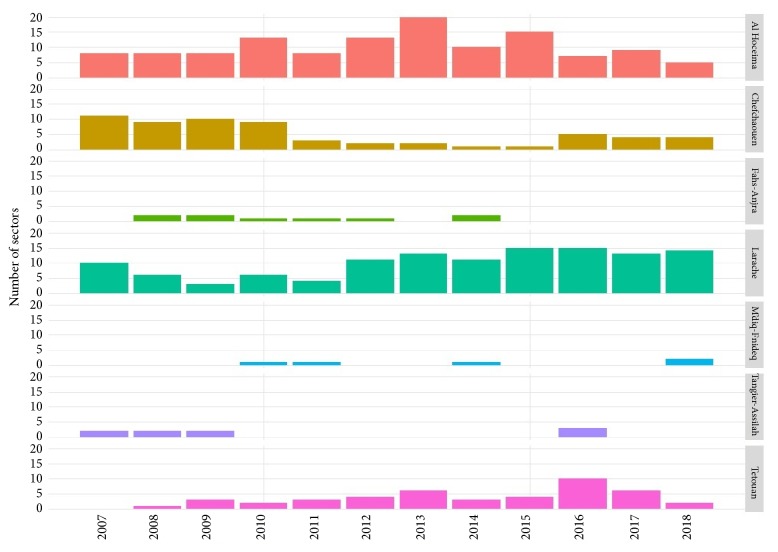
Number of affected sectors by CL depending to years in Tangier-Tetouan-Al Hoceima Region (2007-2018).

Concerning the distribution of cutaneous form, a total of 86 sectors were affected in this region with 65.15% (86/132) (Figure 7). The highest number of affected sectors were observed in Larache Province 100% (n= 19/19) followed by Al Hoceima Province with 77.14% (n= 27/35) of affected sector. About Chefchaouen Province, 58.82% (n= 20/34) of the sectors have been touched.  Figure 8 shows the number of affected sectors by CL in each province per year.

#### 3.2.3. Repartition of CL and VL in Relation to Age and Sex

**Figure 9:**
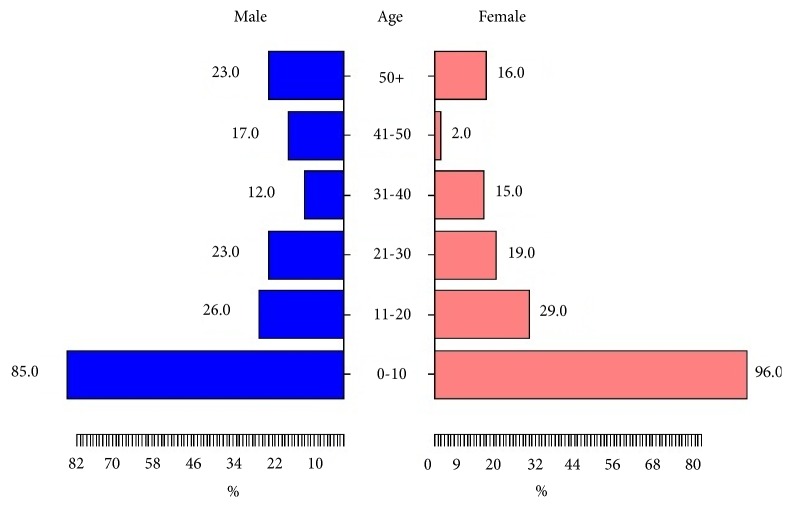
Distribution of leishmaniasis cases in relation to age and sex (2007-2018).

This study was performed to show the most infected population in this region. The statistical study about the distribution of leishmaniasis according to sex has shown there is not a significant difference between genders (Pearson* Chi-square* test:* χ*^*2*^ = 0.22314,* df*= 1,* P* = 0.6367) with a slight predominance of leishmaniasis in males (51.23%* vs.* 48.76% for females; sex-ratio M/F= 1.05). About age group, 49.86% were children under 11 years old. The difference was statistically significant regarding the other age group (Pearson Chi-square test:* χ*^*2*^= 300.82,* df*= 5,* P*<2.2e-16) (Figure 9).

## 4. Discussion

The region of Tangier-tetouan-Al hoceima, made up of seven provinces (Tangier-Assilah, Fahs-Anjra, Tetouan, Larache, Chefchaouen, Al Hoceima, and Mdiaq-Fnidq), is known to be among the moderately affected regions by leishmaniasis especially the cutaneous form. During the period 1997-2018, 1.56% of the reported cases of leishmaniases in Morocco were registered in this region (n= 1,255/80,299). Concerning clinical types, the cutaneous form represented 0.90% (n= 700/78,001) of the reported leishmaniasis cases in Morocco, whereas the visceral form accounted for 24.15% (n= 555/2,298) [32].

In order to properly intervene in the fight against leishmaniasis in this region, a persistent follow-up of this pathology is indispensable, in particular the visceral form, which is considered as a deadly form and which continues to record a rather large number of cases. The measures of control of this disease should take into account rapid diagnosis in suspected clinical cases and treatment of confirmed positive cases, vector control measures, improvement of hygiene conditions, and the zoonotic cycle of this form. Unfortunately, the results showed that these interventions are not yet taken into consideration.

Regarding the molecular characterization, the identification of DNA from CL slides showed that* L. infantum* and* L. tropica* circulate together in this region with a predominance of* L. infantum *(n = 68/81; 84%) (*P*-value = 6.026e-06), while* L. infantum *was the only causal species of VL. On the other hand, the distribution of these parasites showed that* L. infantum *was the only circulating species in Chefchaouen, Tangier-Assilah, and Fahs-Anjra provinces, while in Larache, Al Hoceima, and Tetouan, the predominance of* L. infantum* beside* L. tropica* was registered. Indeed, this is the first identification of this species as the main causative agent of human cutaneous form of leishmaniasis in this region.

The dominance of* L. infantum* is no longer surprising. In fact, this species starts to have a wide extension throughout the world, including several Mediterranean countries, in particular Southern Europe, such as the famous foci in Abruzzi (Italy) [34], and Cukurova region (Turkey) [35]. Also several other epidemiological studies were performed in North Africa such as Tunisia and Algeria [36, 37].

In Morocco this species was responsible for sporadic cases in the north of the country [4]. Indeed, the molecular survey of leishmaniasis in Taza Province showed the existence of many cases of CL due to* L. infantum *[23]. The same results were observed in another study realized in Ouazzane and Sid Kacem Provinces [25]. Going further, exactly to the south of the country, a sporadic case of CL due to* L. infantum* in Ouarzazate Province was declared [38].

The presence of* L. infantum* in this region at a high rate could be explained firstly by the dominance of VL due to* L. infantum* in this area. On the other hand, our study area belongs to humid, subhumid, and semiarid climate located at an altitude of between 0 and 573m. Indeed, Rioux* et al*. in 1984 showed that the repartition of different sandflies species is mostly related to the bioclimatic areas [39]. In addition, Laqraa* et al.* in 2015 provided an updated distribution of leishmaniasis vectors in Morocco according to their bioclimatic and altitudinal preferences [33]. This update showed an abundance of sandfly species known to be vectors of VL and CL in this region. These species include* Phlebotomus perniciosus,* which dominates in the humid, subhumid and semiarid zones at high altitudes and* Phlebotomus longicuspis* which is preponderant in semiarid stages at low altitudes [33, 40]. This may explain the abundance of this* Leishmania* species in the north part of the country, which is characterized by this type of climate.

The epidemiological study established during the surveyed period showed that peaks in the number of human CL cases were recorded in three provinces (Larache, Al Hoceima, and Chefchaouen). The number of cases continues to increase in the provinces of Larache and Al Hoceima, while a remarkable decrease is known in Chefchaouen Province. Moreover, Tetouan Province has recorded a lower number. However, the epidemiological situation of the visceral form showed an important geographical extension, especially in Chefchaouen and Al Hoceima Provinces. Several factors can explain the increase of the number cases until 2010. Among these factors, the active screening is carried out following the introduction of a response action plan between 2010 and 2016. In addition, this increase can be explained also by the neighbourship of these provinces with several provinces already known to be foci of CL and VL such as Ouazzane [25], Sidi Kacem [25], Taounate [41], and Taza [23]. Also, the population activities and shifting could also cause this increase [42]. Moreover, the majority of these cities are located on a mountainous area that is surrounded by old unrestored fissured walls, with a nearby spring that provide daytime resting places for sandflies.

Furthermore, most cases have been reported in rural and periurban areas. In fact, the provinces of Chefchaouen, Al Hoceima, and Larache, which recorded the maximum number of cases, have a relatively low rate of urbanization (respectively, 12.5%, 32.5%, and 52.5%) [26]. According to the WHO, the urbanization rate is indicated as a key factor in the increase of leishmaniasis [43]. The transmission of leishmaniasis generally occurs in rural areas [44], in which it could be related to human behavior through human-animal coexistence and the accumulation of animal waste near homes [45]. Boussaa et al. have confirmed that this factor has a huge influence on vector populations and consequently on the epidemiology of the disease. The abundance of sandflies appears to decrease with increasing urbanization and some potential vector species may disappear [46]. In addition, movement population from rural neighboring foci to periurban areas may increase leishmaniasis cases which are due to the poor quality of life and socioeconomic status [47, 48]. These factors constitute favorable conditions for the propagation of reservoir hosts and vectors and consequently for the spread of leishmaniasis [47].

According to the clinical study of leishmaniasis in Tangier-Tetouan-Al Hoceima Region, the repartition of CL and VL in relation to age showed that no age group was spared from leishmaniasis with the dominance of children under 9 years old. This dominance could be explained by the weak immune system and consequently the inability to fight the* Leishmania* infection. In addition, this may be due also to the habits of children who often play near breeding sites which make them prone to insect bites [49]. Moreover, this study also shows a predominance of men with leishmaniasis; this dominance could be explained essentially by the rural character of the provinces where people's activities depend closely to the breeding generally practiced by men.

In addition, the predominance of this cutaneous form due to* L. infantum* prompts us to know whether an immune suppression of the patients with this form can lead to relapses and develop a visceral form of leishmaniasis. Indeed this species proved to be an important opportunistic agent with high rates of relapse and death in patients with acquired immunodeficiency [50]. Moreover, the emergence of an anthroponotic cycle of HIV/VL coinfections by sharing contaminated syringes among intravenous drug users has been reported [51]. Additionally, it is important to note that the provisional number of cumulative cases of HIV-AIDS in Morocco was 12,545 in 2016 according to the Ministry of Health [52].

Molecular identification of circulating species of* Leishmania* and knowledge of temporal and spatial distribution of leishmaniasis cases are essential in order to understand epidemiology of the diseases [53].

In fact, the transmission cycle of* L. infantum* is zoonotic; dogs have been implicated as the main reservoir hosts of this species. In Morocco, both the MON-1 and MON-24 zymodemes have been isolated from dogs [10, 54]. However, data on density of dogs and their positivity for* L. infantum* in this region are missing. Furthermore, it is important to mention that rodents may also transmit this parasite of which* L. infantum* has been isolated from rodents belonging to the species* Rattus norvegicus* in Greece and Brazil [55, 56].

Interestingly, the identification of* L. infantum* species causing human cutaneous form in this region will play a major role in helping and guiding the national leishmaniasis control program by preventing and taking into consideration the zoonotic character.

## 5. Conclusion

The present study concerns both cutaneous and visceral leishmaniasis prevalence in northern Morocco. Our results showed that two* Leishmania *species* (L. infantum* and* L. tropica)* are present in the northern region of Morocco with a predominance of* L. infantum*. These findings are consistent with studies which have shown that* L. infantum* is the main agent responsible for VL and CL cases in the Mediterranean subregion.

The identification of circulating zoonotic* L. infantum *species in this region is of great importance since it allows the determination of transmission cycles. In fact, these funding will allow us to monitor the health of human and animal with thinking about “One health” as the potential of closer cooperation between human and animal health.

## Figures and Tables

**Table 1:** Molecular results of cutaneous and visceral leishmaniasis slides from the most.

Province	Sector	Urban/Rural	Cutaneous form		Visceral form	Total
			*L. infantum*	*L. tropica*	*L. infantum*	
Larache	Ben said	R	2	0	-	2
	Boujedyane	R	1	3	-	4
	Bghadda	R	2	0	-	2
	Zouada	R	1	0	-	1
	Od Khalkhal	R	1	0	-	1
	Sahel	R	1	0	-	1
	Larache Center	U	2	0	-	2
	Maada	R	1	0	-	1
	DharRouah	R	1	0	-	1
	Ayacha	R	4	0	-	4
	Riassana	R	5	0	-	5
	Od harti	R	1	0	-	1
	Zaaroura	R	1	0	-	1
	Souk Toulba	R	2	0	-	2
	Ksar Kbir	U	2	0	-	2
	Souk L'qolla	R	1	0	-	1
	Laouamra	R	1	0	-	1
	Bni Arouss	R	2	0	-	2
	Al Manar	R	1	0	-	1
	Ksar Bjir	R	1	0	-	1
	Bni Garfet	R	1	1	-	2

Al Hoceima	Ajdir	U	0	1	-	1
	Imzouren	R	5	0	1	6
	Ait Youssef Ouali	R	1	0	-	1
	Bni Bouayach	R	3	0	-	3
	Taghzout	R	0	1	-	1
	Arbaa Taourirt	R	1	0	-	1
	Bni Abdellah	R	1	0	-	1
	Izemmouren	R	1	0	-	1
	Al Hoceima Center	U	1	0	-	1
	Senada	R	1	0	-	1
	Boudinar	R	1	0	-	1
	Arbaa Taourirt	R	1	0	-	1
	Nekkour	R	2	0	-	2
	Targuist	R	1	0	-	1
	Anzagh	R	-	-	1	1
	Kalabonita	R	-	-	1	1
	Douar Assammar	R	-	-	1	1
	Ait ziane	R	-	-	1	1

Tetouan	Zinate	R	1	3	-	4
	Azla	R	0	1	-	1
	Bni Hsen	R	0	1	-	1
	-		1	0	-	1
	Tetouan	U	2	0	-	2
	-		1	0	-	1
	-		-	-	1	1

Chefchaouen	-	-	3	0	-	3
	Stehat	R	1	0	-	1
	Chefchaouen Center	U	0	1	-	1

Fahs Anjra	Anjra	R	1	0	-	1
	Khmis Anjra	R	1	0	-	1
	-		1	0	-	1

Tanger-Assilah	Gzenaya	U	1	0	-	1
	-		1	0	-	1
	-		-	-	2	2

Mdiq-Fnidq	Mdiq	U	1	1	-	2

R: rural, U: urban.

**Table 2:** *Leishmania* species responsible for cutaneous leishmaniasis in the northern region.

Provinces	*L. infantum*	*L. tropica*	Total	*P*-value
Larache	34	4	38	1.135e-06∗
Al Hoceima	19	2	21	0.00020∗
Tetouan	5	5	10	1
Chefchaouen	4	1	5	-
Fahs Anjra	3	0	3	-
Tanger-Assilah	2	0	2	-
Mdiq-Fnidaq	1	1	2	-
Total	68	13	81	9.894e-10*∗*

∗ *P*< 0.001.

## Data Availability

The data used to support the findings of this study are included within the article.

## Abbreviations

CL: Cutaneous leishmaniasis
VL: Visceral leishmaniasis
NRLL: National Reference Laboratory of Leishmaniasis-Rabat
NIH: National Institute of Hygiene-Morocco.
